# Segment-Level Evaluability of Dedicated Non-ECG-Gated Coronary CT Angiography: Associations with Calcification, Heart Rate, Vessel Territory, and Caliber

**DOI:** 10.3390/diagnostics16193234

**Published:** 2026-10-07

**Authors:** Kun Wang, Aiyun Sun, Xia Qiu, Chunqiao Wu, Hong Ren, Yueqiao Zhang

**Affiliations:** 1 Department of Radiology, Sir Run Run Shaw Hospital, Zhejiang University School of Medicine, Hangzhou 310000, Chinawucq@zju.edu.cn (C.W.); 2 CT Imaging Research Center, GE HealthCare, Shanghai 200000, China

**Keywords:** coronary CT angiography, non-ECG-gated CT, coronary calcification, motion artifact, image quality, segment evaluability

## Abstract

**Background/Objectives:** Dedicated coronary CT angiography (CCTA) without physical electrocardiographic gating may simplify acquisition, but segment-level interpretability may vary across the coronary tree. We evaluated coronary segment evaluability and factors associated with non-evaluability on dedicated non-ECG-gated CCTA. **Methods:** This retrospective single-center study included 320 patients and 3739 anatomically present coronary segments with a reference diameter of at least 1.5 mm. Evaluability required confident classification of diameter stenosis as <50% or ≥50%. Segment calcification was graded separately using a prespecified visual scale as none, mild, moderate, or severe. Patient-clustered generalized estimating equations used a binomial distribution, logit link, exchangeable working correlation, and robust standard errors. **Results:** Overall, 3378 segments (90.3%) were evaluable. All included segments were evaluable in 118/320 patients (36.9%); 202/320 (63.1%) had at least one non-evaluable segment and 111/320 (34.7%) had at least one proximal/mid non-evaluable segment. Examination success was 320/336 (95.2%). When 182 observed eligible segments from the 16 globally non-diagnostic examinations were included as non-evaluable, segment evaluability was 3378/3921 (86.2%); an additional 18-segment worst-case boundary yielded 3378/4027 (83.9%). Evaluability declined from 93.4% without calcification to 88.8%, 84.3%, and 74.3% with mild, moderate, and severe calcification. Adjusted odds ratios for non-evaluability were 1.95 (95% CI, 1.46–2.60), 3.13 (2.18–4.49), and 6.53 (4.39–9.71), respectively, versus no calcification. Higher heart rate (per 10 beats/min: OR, 1.52; 95% CI, 1.36–1.69) and right coronary artery location (OR, 1.56; 95% CI, 1.20–2.02) were associated with non-evaluability, whereas larger reference diameter was associated with lower odds (per 1 mm: OR, 0.69; 95% CI, 0.53–0.89). **Conclusions:** Most included coronary segments were evaluable on dedicated non-ECG-gated CCTA, but interpretability varied with calcification, heart rate, vessel territory, and caliber. These findings characterize segment-level feasibility and do not establish diagnostic accuracy, equivalence to ECG-gated CCTA, or a clinical eligibility threshold.

## 1. Introduction

Coronary CT angiography is conventionally performed with ECG synchronization so that data acquisition or reconstruction can be aligned with periods of relatively low coronary motion. This strategy improves temporal consistency and reduces motion artifact, but it also requires ECG setup and, depending on acquisition mode, may constrain scan timing and reconstruction options [1].

Recent advances in CT hardware and reconstruction techniques have progressively reduced the dependence of coronary CT angiography (CCTA) on conventional electrocardiographic (ECG) synchronization. Wide-detector coverage, improved temporal resolution, automated phase selection, and dedicated coronary motion-correction algorithms can reduce motion-related degradation and broaden the range of cardiac conditions under which coronary arteries can be assessed. More recently, ECG-less or non-ECG-gated coronary imaging strategies have been explored, in which coronary reconstruction is performed without physical ECG-lead synchronization. Instead, surrogate cardiac timing, automated phase identification, and motion correction are combined to identify reconstructions with reduced residual coronary motion. Importantly, this concept differs from opportunistic coronary assessment on routine chest CTA: the examination remains a coronary-focused angiographic study with coronary-specific scan range, contrast timing, thin-section reconstruction, and dedicated coronary post-processing. In addition to simplifying patient preparation, elimination of ECG-lead placement may streamline examination workflow when conventional ECG-gated preparation is impractical or time-sensitive [2,3,4,5,6,7].

Emerging evidence supports the technical feasibility of this approach. Previous studies have demonstrated that dedicated ECG-less CCTA can provide diagnostically interpretable coronary images [2,6] and, in selected populations, diagnostic performance against invasive coronary angiography [2]. Zhao et al. recently reported 100% patient-level interpretability with whole-heart motion correction in CCTA performed without ECG leads, with interpretability of 99.6% in proximal/mid segments and 97.2% in distal/small-branch segments [6]. A recent prospective feasibility study by Thomsen et al. further demonstrated the technical feasibility of coronary CT angiography performed without conventional ECG synchronization [7]. Motion-correction strategies have also been evaluated on other CT platforms; Wang et al. reported particularly relevant improvement in right coronary artery image quality at heart rates ≥75 beats/min using a different vendor-specific cardiac motion-correction algorithm [8]. Nevertheless, available studies have largely emphasized overall image quality, reconstruction comparisons, examination-level interpretability, or diagnostic accuracy in selected cohorts. The determinants of failure at the individual coronary-segment level remain less well characterized. This distinction is clinically relevant because an examination may appear acceptable overall while a single clinically important segment remains non-evaluable.

Several factors may be particularly important in the absence of conventional ECG synchronization. Coronary motion varies by vessel and increases with heart rate, potentially affecting the right coronary artery disproportionately [9,10]. Calcification-related partial-volume effects and blooming may obscure the residual lumen, whereas small vessel caliber and distal or branch location may further reduce tolerance to residual motion and image noise [11]. These mechanisms may coexist within the same segment, potentially reducing evaluability despite otherwise acceptable examination-level image quality. Emerging technologies such as photon-counting detector CT offer higher spatial resolution, reduced electronic noise, and improved material discrimination that may benefit coronary imaging, particularly in heavily calcified disease; however, their role in ECG-independent coronary imaging and their ability to overcome motion-related segment failure remain to be established [12]. Defining the practical boundary of current ECG-less CCTA therefore requires analysis at the coronary-segment level rather than reliance solely on overall examination quality.

Therefore, the purpose of this study was to evaluate segment-level diagnostic evaluability in a consecutive cohort undergoing dedicated non-ECG-gated CCTA and to determine the associations of calcification severity, heart rate, coronary territory, segment location, and reference vessel diameter with segment non-evaluability. We hypothesized that non-evaluability would be associated with greater calcification severity, higher heart rate, smaller vessel caliber, and anatomically more motion-sensitive coronary segments.

## 2. Materials and Methods

### 2.1. Study Design and Population

This retrospective, single-center study was conducted in accordance with the Declaration of Helsinki and its subsequent amendments and was approved by the Institutional Review Board of Sir Run Run Shaw Hospital, Zhejiang University School of Medicine (No. 2025-2258-01). The requirement for written informed consent was waived because of the retrospective nature of the study.

Consecutive adult patients who underwent dedicated non-electrocardiography (ECG)-gated coronary computed tomography angiography (CCTA) for the evaluation of suspected coronary artery disease between June 2025 and April 2026 were retrospectively screened. All examinations were performed using a dedicated coronary CTA protocol without placement of physical ECG leads or conventional ECG-triggered data acquisition.

Patients were eligible for inclusion if the acquired CCTA dataset provided complete coverage of the coronary artery tree and was available for dedicated coronary reconstruction and segment-level image-quality assessment. The exclusion criteria were as follows: (I) age <18 years; (II) previous coronary artery bypass grafting; (III) extensive coronary stent implantation precluding reliable assessment of the native coronary lumen; (IV) incomplete coverage of the coronary arteries; and (V) incomplete imaging or clinical data required for analysis. Globally non-diagnostic examinations were not included in the primary segment-level cohort but were retained as patient-level failures for sensitivity analyses.

Importantly, patients were not excluded solely because of a high coronary calcium burden, because the effect of coronary calcification on segment-level evaluability constituted one of the primary objectives of the present study. Similarly, individual coronary segments affected by severe calcification or motion artifacts were retained and classified according to their evaluability rather than excluded from the analysis.

**Figure 1:**
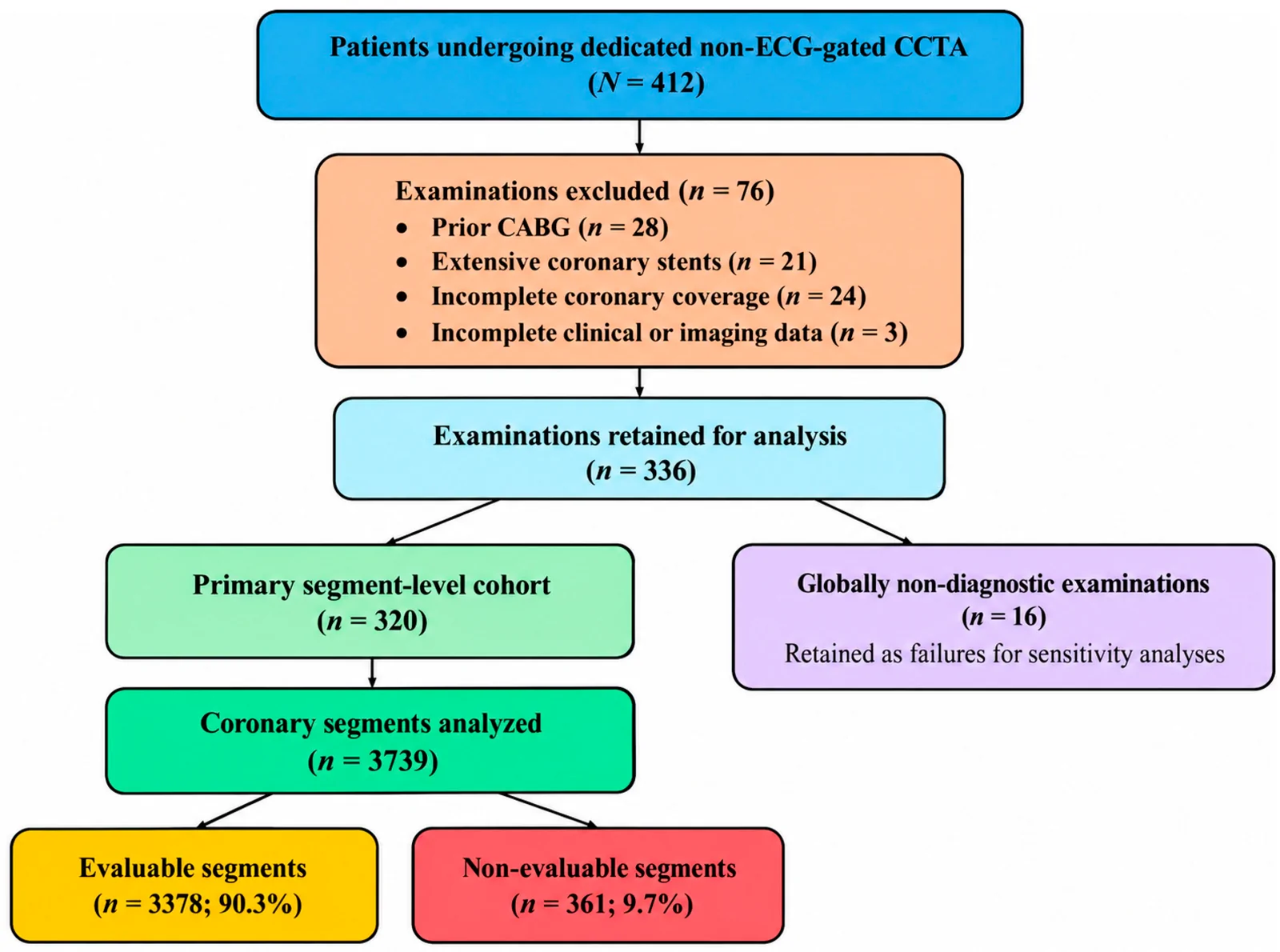
Study flowchart. Of 412 examinations screened, 76 were excluded before analysis: prior CABG (*n* = 28), extensive coronary stents (*n* = 21), incomplete coronary coverage (*n* = 24), and incomplete clinical or imaging data (*n* = 3). A total of 336 examinations were retained for analysis; 320 entered the primary segment-level cohort and 16 globally non-diagnostic examinations were retained as failures for sensitivity analyses. CABG, coronary artery bypass grafting; CCTA, coronary computed tomography angiography.

During the study period, dedicated non-ECG-gated acquisition was the routine institutional CCTA protocol on this CT system rather than a protocol selectively assigned on a patient-by-patient basis. After initial screening, 336 examinations were retained for analysis. Of these, 320 examinations entered the primary segment-level analysis, whereas 16 globally non-diagnostic examinations were retained as patient-level failures for sensitivity analyses. The patient selection process is summarized in Figure 1.

There was no patient overlap between the present cohort and the cohorts reported in Refs. [2] or [4]. Some patients subsequently underwent invasive coronary angiography as part of clinical care; however, no ICA examination performed within the prespecified CCTA–ICA interval of ≤3 months met the matching requirements for inclusion as a diagnostic reference-standard dataset. Therefore, no ICA-based diagnostic-accuracy subgroup was analyzed.

### 2.2. CT Acquisition and Image Reconstruction

All examinations were performed using a 256-row wide-detector CT scanner (Revolution Apex Expert, GE HealthCare, Waukesha, WI, USA) with a dedicated non-ECG-gated coronary CTA acquisition protocol. No physical ECG leads were attached to the patients during image acquisition. Tube voltage was automatically selected from 80, 100, or 120 kV according to patient characteristics and scout images, and automatic tube current modulation was applied with a preset noise index of 20 Hounsfield units (HU). The acquisition window was set to 300–500 ms, with other acquisition parameters including a gantry rotation time of 0.23 s, and scan coverage encompassing the entire heart and coronary artery tree.

Heart rate (HR) was recorded immediately before image acquisition using pulse monitoring. No physical ECG trace was recorded during the scan. The pre-scan pulse-derived heart rate was used to initialize a surrogate cardiac-cycle timing model for virtual phase assignment; the 300–500 ms setting defined the reconstruction search window rather than ECG-triggered acquisition padding. Candidate reconstructions within this virtual window were evaluated by Smart Phase (GE HealthCare), which selected the phase with the least apparent coronary motion [2]. Because the surrogate timing model was based on the pre-scan heart rate, beat-to-beat changes or rhythm irregularity during acquisition could produce phase mismatch; no contemporaneous ECG signal was available to correct such mismatch. Residual coronary motion was subsequently addressed, where possible, using the second-generation coronary motion correction algorithm SnapShot Freeze 2 (SSF2, GE HealthCare), which incorporates information from adjacent reconstructed phases to improve coronary vessel delineation [3].

Iodinated contrast medium (Iopromide, 370 mg I/mL; Bayer, Leverkusen, Germany) was administered intravenously according to a weight-based protocol (approximately 0.6 mL/kg, protocol range 30–60 mL) over 10 s. The mean contrast volume was 42.1 ± 6.4 mL (range, 30–60 mL), and the mean injection rate was 4.21 ± 0.64 mL/s. Patient body weight, administered contrast volume, and injection rate were obtained from the examination records archived in the PACS. An equal-volume saline flush followed at the same flow rate. No β-blockers or nitroglycerin were administered before or during CCTA.

Images were reconstructed with a slice thickness and interval of 0.625 mm using a deep-learning image reconstruction algorithm (TrueFidelity, GE HealthCare) [13]. SSF2-corrected images were used for coronary artery assessment. Reconstructed images were transferred to a dedicated workstation for subsequent analysis using axial images, multiplanar reformations, curved planar reformations, and maximum-intensity projections as appropriate. Smart Phase, SSF2, and TrueFidelity were standard vendor-provided software functions available on the clinical CT system (version 23MW24.39; GE HealthCare, Milwaukee, WI, USA) and were not investigational or custom research software.

### 2.3. Coronary Artery Analysis and Segment Evaluability

Segment-based interpretation is recommended in SCCT reporting guidance [14]. Two cardiovascular radiologists with 7 and 10 years of cardiac CT experience independently evaluated all datasets. The coronary tree was assessed according to the 18-segment SCCT model. Anatomically absent segments and segments with a reference diameter <1.5 mm were excluded. Anatomically present segments degraded by motion, calcification, insufficient enhancement, noise, or other artifacts remained in the denominator and were classified as non-evaluable. For territory analyses, LM was analyzed separately; LAD territory included the LAD and diagonal branches, LCX territory included the LCX, obtuse marginal, and left-sided posterolateral branches, and RCA territory included the RCA, posterior descending artery, and right-sided posterolateral branches. A ramus intermedius, when present, was assigned to the LCX territory for the prespecified territory analysis. For location analyses, named proximal and mid main-vessel segments were grouped as proximal/mid; distal main-vessel segments and side branches were grouped as distal/branch.

Among the 3739 segments included in the primary analysis, reference diameter was measurable in all segments (unmeasurable diameter, n = 0), and all met the prespecified ≥1.5-mm criterion. A total of 464 anatomically present segments were excluded because their measured reference diameter was <1.5 mm.

The primary endpoint was segment-level diagnostic evaluability. A coronary segment was considered evaluable when image quality was sufficient to confidently determine whether the segment had <50% or ≥50% diameter stenosis throughout the assessable lumen. A segment was considered non-evaluable when this distinction could not be made reliably because of motion, calcification-related artifacts, insufficient contrast enhancement, excessive image noise, or other substantial artifacts. Thus, evaluability reflected the ability to assess potentially clinically relevant coronary stenosis rather than the presence of image artifacts alone. The 50% diameter-stenosis boundary is consistent with the distinction between non-obstructive and potentially obstructive disease in standardized CCTA reporting; the present evaluability endpoint nevertheless reflects reader confidence rather than independently verified diagnostic accuracy [15]. Although evaluability was defined by whether a segment could be confidently classified as <50% versus ≥50% stenosis, the actual stenosis category was not recorded as a structured study variable because diagnostic-accuracy assessment was not a prespecified endpoint. We therefore could not provide a verified stenosis-prevalence or CAD-RADS distribution.

### 2.4. Assessment of Coronary Calcification

Coronary calcification was assessed separately for each coronary segment using axial images and cross-sectional images reconstructed perpendicular to the vessel centerline, with curved planar reformations used when necessary. Calcification severity was determined according to the morphological extent and distribution of calcified plaque within the segment, independent of residual lumen visibility or diagnostic evaluability.

A study-specific visual ordinal scale classified calcification as follows: none, no visually detectable calcification; mild, isolated or focal calcification involving a limited portion of vessel circumference and longitudinal extent; moderate, more extensive calcification involving an intermediate proportion of circumference and/or segment length; and severe, extensive calcification involving a large proportion of circumference and/or a substantial longitudinal portion of the segment. No quantitative Agatston or volumetric cutoffs were applied.

When multiple discrete calcified plaques were present within the same segment, the overall calcification grade was determined by considering their cumulative longitudinal extent together with the greatest circumferential involvement observed within that segment. The higher category was assigned when the circumferential and longitudinal extent suggested different grades.

Lumen obscuration or segment non-evaluability was not used as a criterion for assigning calcification severity. Calcification and evaluability therefore had separate operational definitions; however, because both were assessed by the same readers on the same CCTA datasets, perceptual coupling between calcium blooming and lumen visibility cannot be excluded.

### 2.5. Assessment of Motion Artifacts and Subjective Image Quality

Coronary motion artifacts were assessed independently using a 4-point scale: grade 0, no perceptible motion artifact with sharply defined vessel margins; grade 1, mild vessel-edge blurring without apparent contour duplication; grade 2, moderate blurring or partial double-contour appearance; and grade 3, marked blurring, contour duplication, or apparent vessel discontinuity. Motion grade was assigned according to the morphological appearance of the vessel and was not determined by whether the segment was ultimately considered evaluable.

Subjective image quality was also assessed separately using a 4-point scale: 4, excellent, with sharp vessel margins and minimal or no artifacts; 3, good, with minor image degradation but largely preserved vessel definition; 2, fair, with moderate image degradation and reduced vessel sharpness; and 1, poor, with marked image degradation and substantial loss of vessel definition. Subjective image quality and diagnostic evaluability were recorded as separate variables, and the image-quality score itself did not determine whether a segment was classified as evaluable.

### 2.6. Reference Vessel Diameter and Objective Image Quality

Reference vessel diameter was measured on cross-sectional images reconstructed perpendicular to the vessel centerline. Whenever possible, measurements were obtained from a relatively normal-appearing portion of the corresponding coronary segment without substantial focal stenosis or calcified plaque. When suitable proximal and distal reference sites were both available, the mean of the two measurements was used. If the target portion itself was degraded, the nearest technically measurable reference portion within the same named segment was used; segments for which a reliable reference diameter could not be established were not eligible for the diameter-based analysis. Reference diameter was measured from inner lumen edge to inner lumen edge and recorded in millimeters. The analysis included only segments meeting the prespecified ≥1.5-mm criterion. A total of 464 anatomically present segments were excluded because the measured reference diameter was <1.5 mm.

Objective image quality was assessed by measuring vascular attenuation and image noise in the aortic root. A circular region of interest was placed within the contrast-enhanced blood pool while avoiding the vessel wall, calcification, and visible artifacts. Mean attenuation was recorded in Hounsfield units (HU), and image noise was defined as the standard deviation of CT attenuation within the same region of interest.

### 2.7. Interobserver Agreement

The two readers independently assessed segment evaluability, calcification severity, motion artifact grade, and subjective image quality. Calcification severity and segment evaluability were recorded as separate variables, and lumen visibility was not used as a criterion for calcification grading.

The two readers independently assessed each coronary segment using the same prespecified sequence: calcification severity was assessed first, followed by motion-artifact grade and subjective image quality, and segment evaluability was assessed last. These assessments were performed during the same reading session without a prespecified washout interval. Interobserver agreement for binary segment evaluability was assessed using Cohen’s κ [16], whereas agreement for calcification severity, motion-artifact grade, and subjective image quality was summarized using weighted κ point estimates from the original interobserver analysis [17]. Disagreements in evaluability were resolved by consensus. Consensus assessments were used for the final segment-level analyses and multivariable modeling. For Cohen’s κ, the 95% confidence interval was estimated using patient-level cluster bootstrap resampling with 2000 replicates to account for within-patient clustering of coronary segments. Complete paired segment-level ordinal ratings were not available for recalculation of confidence intervals for the three weighted κ estimates.

### 2.8. Statistical Analysis

Primary statistical analyses were performed using SPSS Statistics version 26.0 (IBM Corp., Armonk, NY, USA). Additional cluster-bootstrap confidence intervals, sensitivity GEE models, and supplementary figures were generated in Python 3.13.5 using statsmodels 0.14.6, SciPy 1.17.0, and Matplotlib 3.10.8. Continuous variables are presented as mean ± standard deviation or median with interquartile range, as appropriate, and categorical variables as numbers and percentages. Segment-level group summaries were treated as descriptive because observations were clustered within patients. For descriptive estimates of segment evaluability according to calcification severity, 95% confidence intervals were obtained using patient-level cluster bootstrap resampling with 2000 replicates to account for within-patient correlation among coronary segments [18].

Because multiple coronary segments were evaluated within each patient, GEE with a binomial distribution and logit link function was used to model segment non-evaluability. Patient identity was the clustering variable, an exchangeable working correlation structure was specified, and robust sandwich standard errors were used. The model included calcification severity, heart rate, body mass index, reference vessel diameter, location, coronary territory, and image noise. Heart rate and body mass index were modeled per 10-beats/min and 5-kg/m^2^ increments; diameter and noise were modeled per 1-mm and 1-HU increments. The analysis dataset contained no missing values for the outcome or model covariates; therefore, all 3739 segments entered the complete-case model. Adjusted odds ratios (ORs) with 95% confidence intervals (CIs) were reported. All tests were two-sided, and *p* < 0.05 was considered statistically significant [18,19]. For descriptive territory analyses, LM was reported separately. In the multivariable GEE model, LM and LAD were combined as the reference territory category because relatively few LM segments were non-evaluable.

Sensitivity analyses counted each globally non-diagnostic examination as a patient-level failure. For these examinations, anatomical presence and reference diameter were assessed on the best available source and reconstructed images. Segments were included in the observed-segment sensitivity analysis only when they could be anatomically identified and a reference diameter ≥1.5 mm could be measured despite the examination-level failure. The 16 globally non-diagnostic examinations contained 182 such observed anatomically present coronary segments; these observed segments were classified as non-evaluable in the sensitivity analysis. As an additional worst-case boundary, all 18 SCCT segments were assigned as non-evaluable to each failed examination. Patient-level endpoints were derived directly from the included segments. Heart rate was grouped as <65, 65–80, and >80 beats/min. Departure from a linear heart-rate association was examined by adding a quadratic heart-rate term to the clustered GEE model. Complete-vessel evaluability required every included segment in that territory to be evaluable. A sensitivity model omitting reference diameter was used to assess coefficient stability when vessel caliber and anatomical location were considered together. The corresponding sensitivity-model forest plot is provided in Figure S2.

## 3. Results

### 3.1. Patient and Scan Characteristics

The cohort comprised 320 patients (mean age 60.7 ± 10.1 years; 183 men [57.2%]). Mean height was 167.1 ± 7.5 cm and mean body weight was 70.1 ± 10.8 kg. The pre-acquisition heart-rate range was 45.0–104.3 beats/min, and mean heart rate before acquisition was 72.9 ± 11.3 beats/min. Rhythm was sinus in 293 patients (91.6%), atrial fibrillation in 15 (4.7%), and frequent ectopy in 12 (3.8%). Mean contrast volume was 42.1 ± 6.4 mL and mean injection rate was 4.21 ± 0.64 mL/s (Table 1). Tube voltage was 80 kV in 52 examinations (16.3%), 100 kV in 221 (69.1%), and 120 kV in 47 (14.7%). All examinations were conducted using non-ECG-gated CCTA.

### 3.2. Patient- and Vessel-Level Evaluability

Among 320 patients in the primary segment analysis, 118 (36.9%) had all included coronary segments evaluable and 202 (63.1%) had at least one non-evaluable segment. A proximal or mid segment was non-evaluable in 111 patients (34.7%). The median number of non-evaluable segments per patient was 1 (IQR, 0–2); 118, 103, 60, and 39 patients had 0, 1, 2, and ≥3 non-evaluable segments, respectively. Complete-vessel evaluability was 94.1% for LM, 75.9% for LAD, 79.4% for LCX, and 60.0% for RCA (Table S1).

### 3.3. Overall Segment Evaluability

A total of 3739 coronary segments from 320 patients met the predefined criteria. Of these, 3378 (90.3%) were evaluable and 361 (9.7%) were non-evaluable (Figure 1).

### 3.4. Impact of Calcification

**Figure 2:**
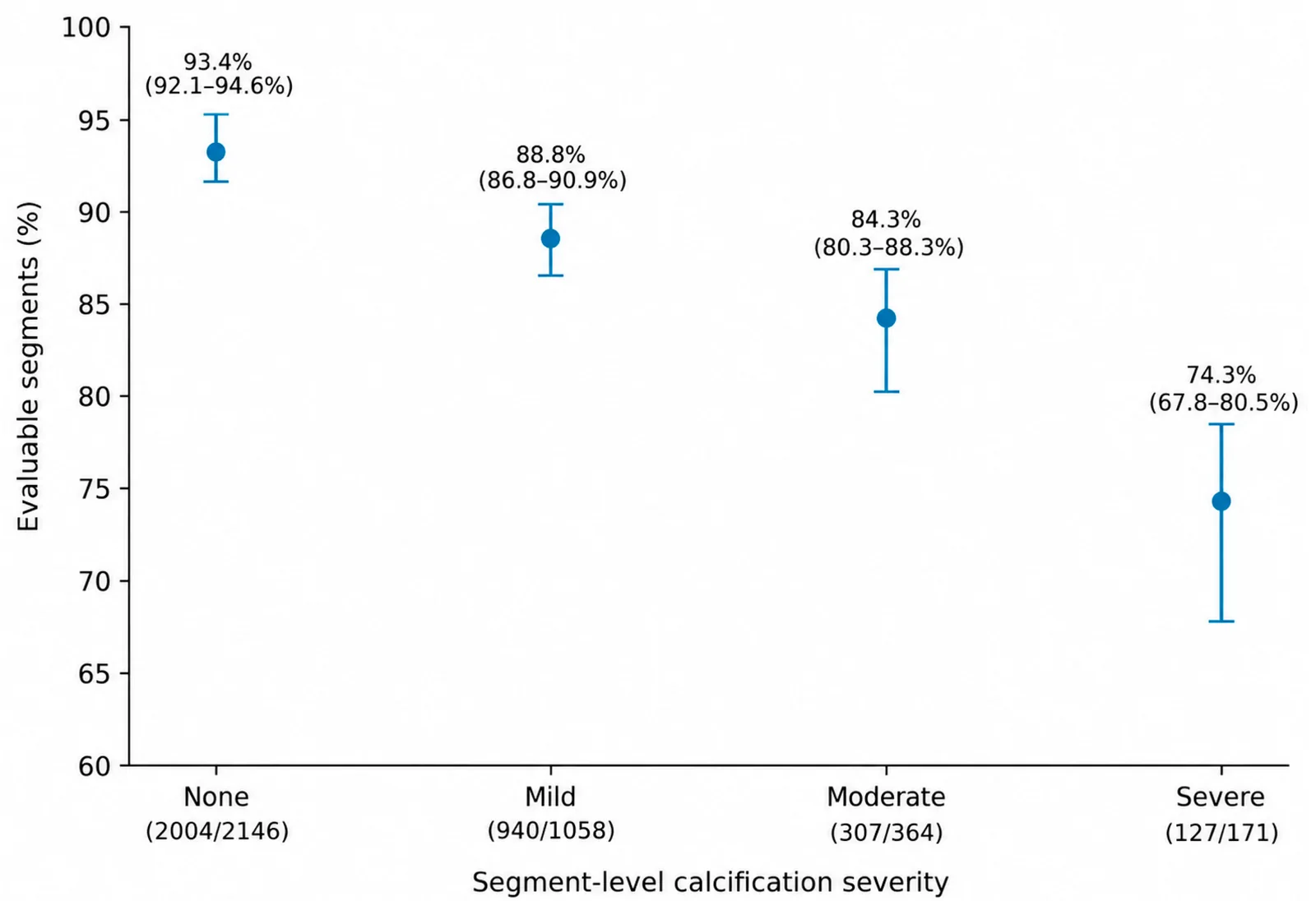
Coronary segment evaluability by calcification severity. Points show evaluable segments as a percentage of all segments in each category; error bars are percentile 95% confidence intervals from patient-level cluster bootstrap resampling (2000 replicates; random seed 20260911). Values above the *x*-axis show evaluable segments/total segments.

**Figure 3:**
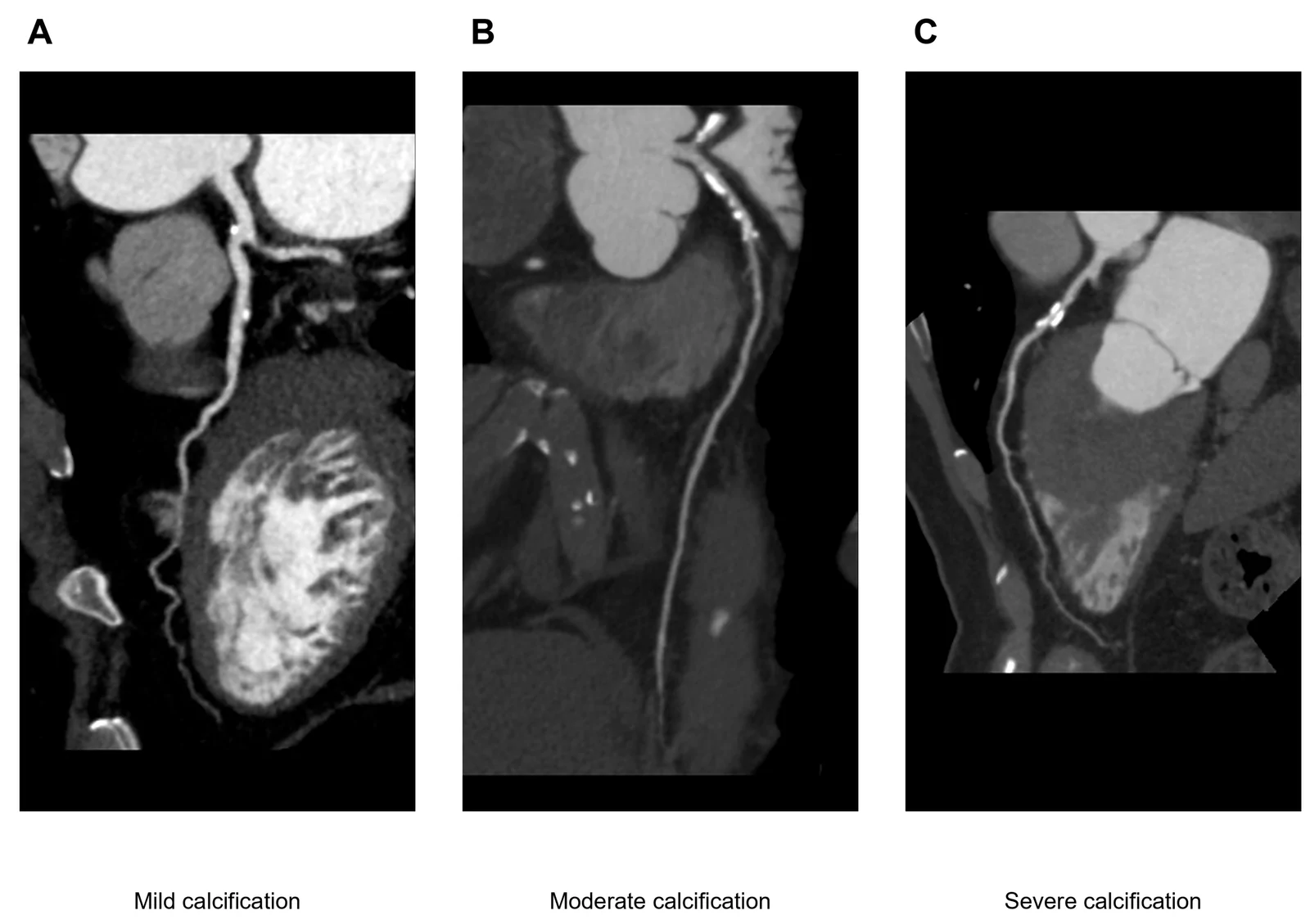
Representative curved planar reformations of evaluable left anterior descending coronary artery segments with (**A**) mild, (**B**) moderate, and (**C**) severe calcification. The three panels are from three different patients. Calcification severity was assigned using the study-specific segment-level visual grading scheme using operational criteria separate from evaluability. Despite increasing calcification burden, the displayed segments remained evaluable for classification of diameter stenosis as <50% or ≥50%.

Segment evaluability decreased progressively with increasing calcification severity, from 93.4% in non-calcified segments to 88.8%, 84.3%, and 74.3% in segments with mild, moderate, and severe calcification, respectively (Table 2 and Figure 2). Representative evaluable LAD segments from three different patients across mild, moderate, and severe calcification categories are shown in Figure 3.

### 3.5. Territory and Segment-Level Characteristics

**Figure 4:**
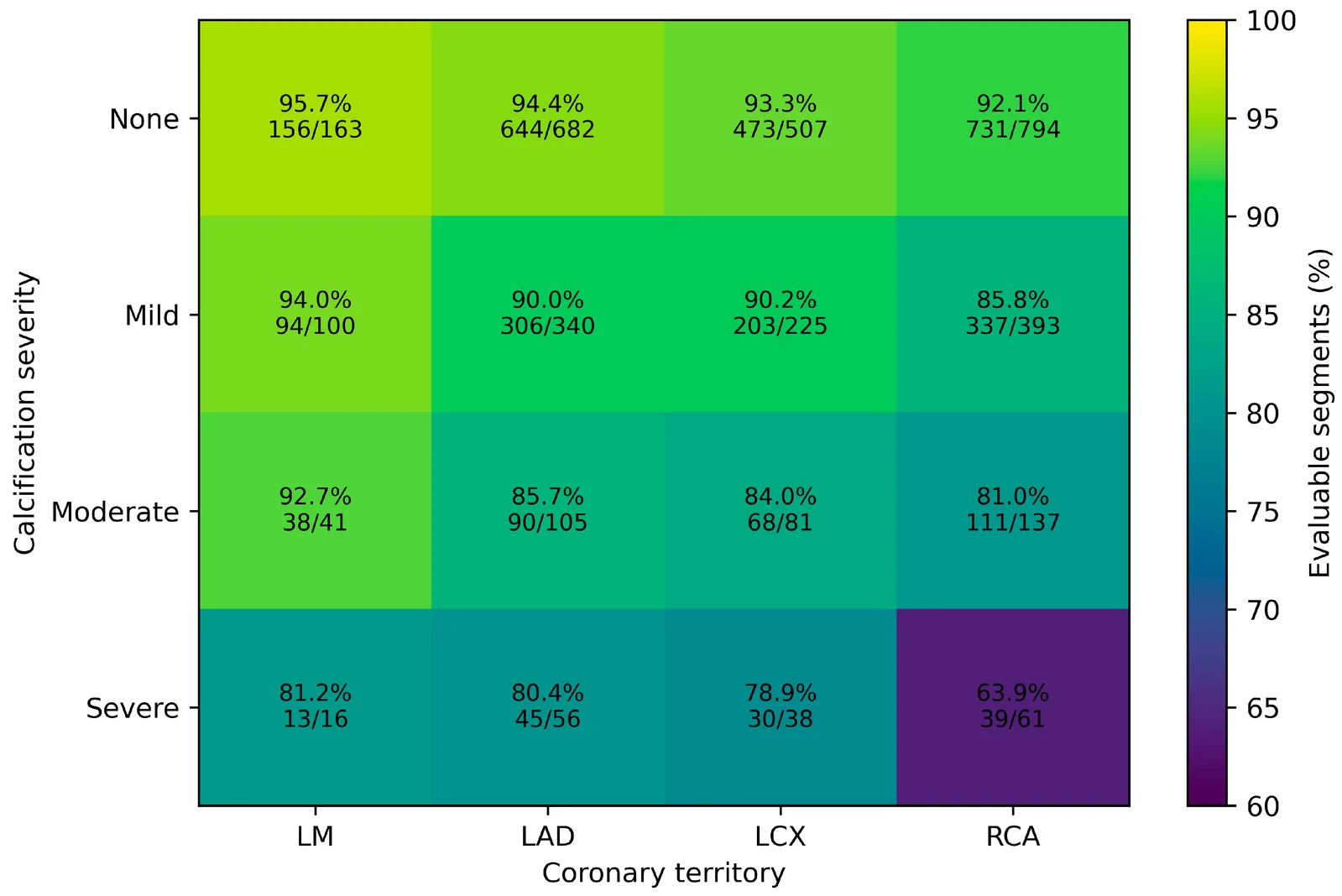
Descriptive heatmap of segment evaluability by calcification severity and coronary territory. Each cell displays the number of evaluable segments/total segments and the corresponding percentage. No interaction hypothesis test was performed; the stratified values are descriptive. LM, left main; LAD, left anterior descending; LCX, left circumflex; RCA, right coronary artery.

Evaluability varied across coronary territories, with the lowest rate observed in the RCA (87.9%). Distal/branch segments also showed lower evaluability than proximal/mid segments (88.1% vs. 92.4%) (Table 3). When calcification severity and coronary territory were considered together, the reduction in evaluability was most pronounced in severely calcified RCA segments (Figure 4).

### 3.6. Interobserver AgreementResults

Reader 1 classified 379/3739 segments (10.1%) as non-evaluable, whereas Reader 2 classified 386/3739 (10.3%) as non-evaluable. Simple agreement for binary segment evaluability was 98.4%, and consensus adjudication was required for 59/3739 segments (1.6%). Interobserver agreement for segment evaluability was excellent (Cohen’s κ = 0.914; patient-cluster bootstrap 95% CI, 0.890–0.935). Weighted κ values from the original interobserver analysis were 0.919 for calcification severity, 0.854 for motion-artifact grade, and 0.902 for subjective image quality. Complete paired segment-level ordinal ratings were not available for recalculation of confidence intervals for these three weighted κ estimates; therefore, only point estimates are reported.

### 3.7. Multivariable GEE Analysis

**Figure 5:**
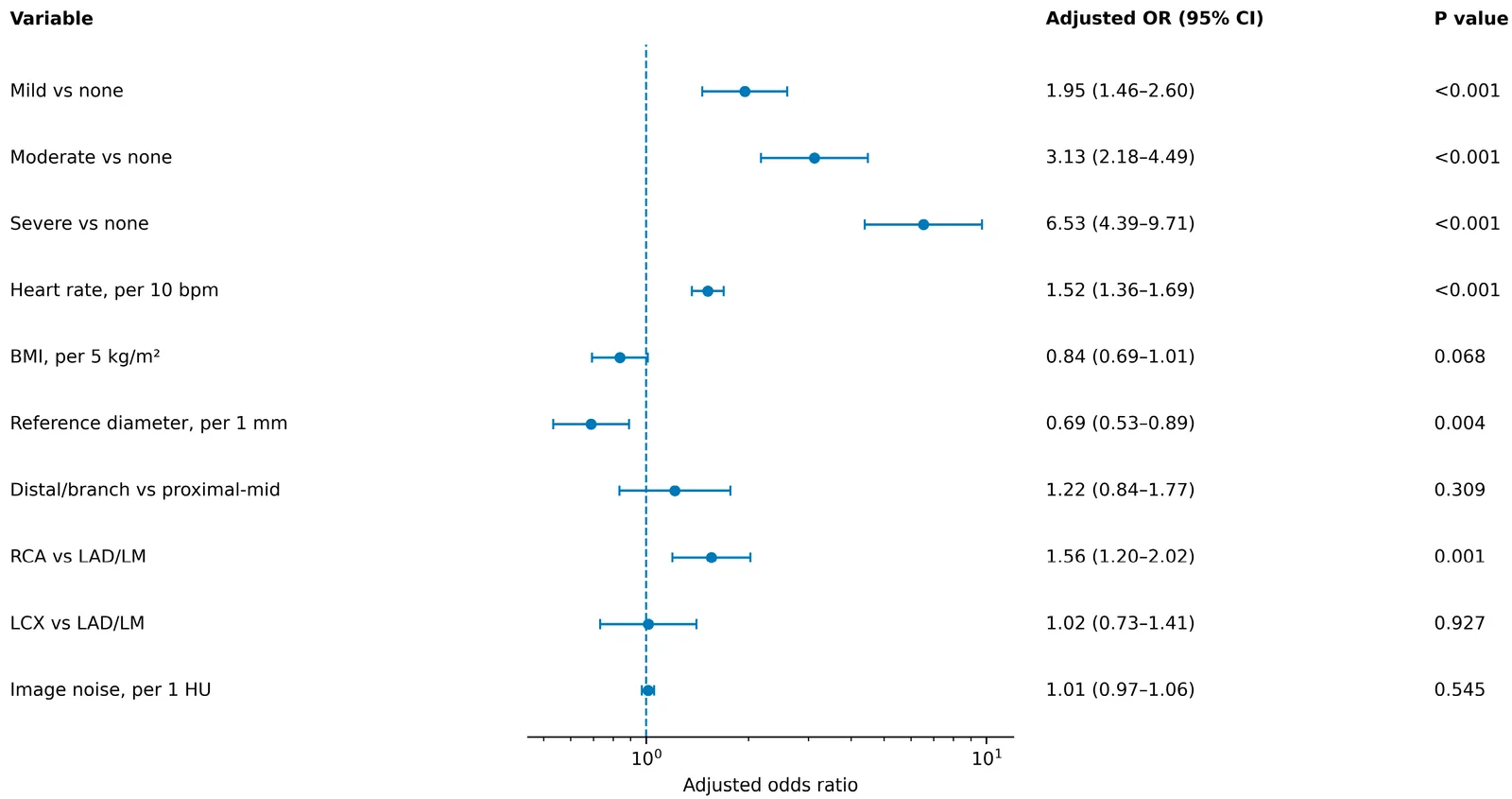
Multivariable predictors of segment non-evaluability. Adjusted odds ratios (ORs) and 95% confidence intervals were estimated using a binomial generalized estimating equation with logit link, patient clustering, an exchangeable working correlation structure, and robust sandwich standard errors. The model was adjusted for segment-level calcification severity, heart rate, body mass index, reference diameter, anatomical location, coronary territory, and image noise. Reference groups were no calcification, proximal/mid location, and LAD/LM territory. OR > 1 indicates higher odds of segment non-evaluability.

Multivariable GEE analysis showed graded associations between calcification severity and segment non-evaluability: mild versus none, OR 1.95 (95% CI, 1.46–2.60; *p* < 0.001); moderate versus none, OR 3.13 (95% CI, 2.18–4.49; *p* < 0.001); and severe versus none, OR 6.53 (95% CI, 4.39–9.71; *p* < 0.001). Higher heart rate (per 10 beats/min: OR, 1.52; 95% CI, 1.36–1.69; *p* < 0.001) and RCA location versus LAD/LM (OR, 1.56; 95% CI, 1.20–2.02; *p* = 0.001) were associated with higher odds of non-evaluability. Larger reference diameter was associated with lower odds (per 1 mm: OR, 0.69; 95% CI, 0.53–0.89; *p* = 0.004). BMI, distal/branch location, LCX territory, and image noise were not statistically significant (Figure 5). In the sensitivity model omitting reference diameter, the associations for calcification, heart rate, and RCA territory were materially unchanged; distal/branch location became significant (OR, 1.85; 95% CI, 1.46–2.35; *p* < 0.001) (Table S3). The corresponding sensitivity-model forest plot is provided in Figure S2.

### 3.8. Heart-Rate and Rhythm Analyses and Sensitivity Analyses Including Globally Non-Diagnostic Examinations

Heart-rate strata included 84, 162, and 74 patients at <65, 65–80, and >80 beats/min, with segment evaluability of 922/987 (93.4%), 1726/1889 (91.4%), and 730/863 (84.6%), respectively (Table S2; Figure S1). Addition of a quadratic heart-rate term did not provide evidence of departure from linearity (*p* = 0.177; Table S3). When the 16 globally non-diagnostic examinations were counted as failures, examination success was 320/336 (95.2%; 95% CI, 92.4–97.0%). These 16 examinations contained 182 observed eligible coronary segments, all classified as non-evaluable; inclusion of these segments yielded an observed-failure sensitivity estimate of 3378/3921 (86.2%; 95% CI, 85.0–87.2%). At the patient level, the 16 failed examinations included 12 patients with sinus rhythm, 3 with atrial fibrillation, and 1 with frequent ectopy; their primary examination-level failure reasons were severe respiratory/bulk motion in 9, inadequate contrast enhancement in 5, and major reconstruction failure in 2 (Table S5). An additional worst-case boundary assigning all 18 SCCT segments as non-evaluable to each failed examination yielded 3378/4027 (83.9%; 95% CI, 82.7–85.0%; Table S3).

Among the primary cohort, segment evaluability was 3085/3421 (90.2%) for sinus rhythm, 159/175 (90.9%) for atrial fibrillation, and 134/143 (93.7%) for frequent ectopy. Across all 336 examinations, global examination failure occurred in 12/305 (3.9%), 3/18 (16.7%), and 1/13 (7.7%) patients, respectively. Of the three globally non-diagnostic examinations in patients with atrial fibrillation, two failed because of severe respiratory/bulk motion and one because of inadequate contrast enhancement. When the 16 globally non-diagnostic examinations were counted as patient-level failures, all included coronary segments were evaluable in 118/336 patients (35.1%), 218/336 (64.9%) had at least one non-evaluable segment, and 127/336 (37.8%) had at least one proximal/mid non-evaluable segment.

### 3.9. Motion, Image Quality, and Reasons for Non-Evaluability

Across all 3739 included segments, motion grades 0, 1, 2, and 3 were observed in 1952 (52.2%), 1139 (30.5%), 450 (12.0%), and 198 (5.3%) segments, respectively. Subjective image-quality scores of 4, 3, 2, and 1 were assigned to 1530 (40.9%), 1588 (42.5%), 443 (11.8%), and 178 (4.8%) segments, respectively. Among the 361 non-evaluable segments, the primary reason was motion artifact in 202 (56.0%), calcification/blooming in 107 (29.6%), insufficient enhancement in 22 (6.1%), excessive image noise in 18 (5.0%), and other artifact in 12 (3.3%). Overall distributions are shown in Supplementary Figures S3 and S4, and distributions by coronary territory and heart-rate category are provided in Supplementary Table S4.

## 4. Discussion

This study provides three principal observations. First, 90.3% of the 3739 included coronary segments were evaluable on dedicated non-ECG-gated CCTA. Second, calcification showed a graded association with non-evaluability after adjustment. Third, higher heart rate and RCA location were associated with higher odds of non-evaluability, whereas larger reference vessel diameter was associated with lower odds. These findings describe segment-level image interpretability and do not establish stenosis-detection accuracy or validated clinical eligibility thresholds.

The overall evaluability observed in this study supports the technical feasibility of dedicated non-ECG-gated CCTA under the acquisition and reconstruction conditions used. Previous studies have shown that coronary images can be obtained without physical ECG synchronization when wide-detector acquisition, retrospective phase estimation, and motion correction are available [2]. Wang et al. reported comparable subjective image quality and signal- and contrast-to-noise ratios between ECG-less and ECG-gated groups, with a shorter examination time for ECG-less acquisition [4]. Asadian et al. likewise found broadly comparable image quality, although LCX and RCA scores were lower with quarter-cycle ECG-less reconstruction [5]. Ref. [2], from a previous, non-overlapping cohort at our institution, reported patient-level sensitivity of 92.1%, specificity of 91.5%, and 6.4% non-diagnostic segments against invasive coronary angiography; differences in study population and diagnostic-accuracy endpoint limit direct comparison with the present 9.7% segment non-evaluability rate [2]. Zhao et al. reported 100% patient-level interpretability with SSF2 in 70 patients undergoing CCTA without ECG leads, with interpretability of 99.6% in proximal/mid segments and 97.2% in distal/small-branch segments [6]. These higher rates may reflect differences in study population, reconstruction comparison, and endpoint definition. Wang et al., using a different vendor-specific cardiac motion-correction algorithm, found that the greatest benefit occurred in the RCA at heart rates ≥75 beats/min, which is directionally consistent with the present associations of higher heart rate and RCA territory with non-evaluability; however, their exclusion of patients with Agatston scores >400 and different platform limit direct quantitative comparison [8]. The present study extends these examination-level observations by identifying segment characteristics associated with failure. This distinction matters because a study may appear acceptable overall while individual clinically relevant segments remain non-evaluable.

Calcification showed the largest category-specific odds ratio in this model; however, odds ratios for predictors expressed on different scales should not be interpreted as a direct ranking of importance. Evaluability declined progressively across the four calcification categories, while adjusted odds of non-evaluability increased from mild to moderate and severe disease. Calcification and evaluability were defined using separate criteria, so the outcome was not formally used to define the exposure; nevertheless, same-reader assessment and calcium blooming may have introduced correlated measurement error or differential misclassification. The finding is technically plausible: partial-volume averaging and blooming can enlarge the apparent extent of calcium and obscure the adjacent enhanced lumen [11]. Recent angiography-referenced studies continue to show that extensive calcification challenges conventional CCTA, while ultra-high-resolution photon-counting CCTA can reduce calcium blooming; these diagnostic-accuracy endpoints differ from the visual grading and evaluability endpoint used here [20,21]. The descriptive heatmap further suggested particularly low evaluability in severely calcified RCA segments. This pattern may reflect the combined effects of calcium blooming and residual coronary motion, but the small severe-calcification subgroups precluded a formal interaction claim.

Higher heart rate and RCA location provided information beyond calcification severity. Faster heart rates shorten relatively motion-free portions of the cardiac cycle, [9] and the RCA is especially susceptible to cardiac motion [8]. Although motion-correction methods can improve coronary image quality, residual motion may persist under challenging acquisition conditions; the cited studies do not establish a uniform heart-rate-dependent effect for second-generation correction [3,22,23]. Larger reference vessel diameter was protective, consistent with the greater proportional impact of motion blur, partial-volume averaging, and calcium blooming in small vessels [10]. Super-resolution deep-learning reconstruction has improved coronary sharpness and image noise compared with conventional reconstruction, supporting the technical importance of spatial resolution in smaller vessels [24]. Distal/branch location was associated with lower unadjusted evaluability but was not independently significant after adjustment, suggesting that vessel caliber and related imaging characteristics may explain part of the apparent distal-segment disadvantage.

BMI and aortic-root image noise were not independently associated with non-evaluability. Tube-current adaptation and deep-learning reconstruction may have reduced noise-related variation in this cohort [1,13], but the result should not be interpreted as evidence that body habitus or noise is unimportant in CCTA generally. Scanner output constraints can still impair image quality in overweight or obese patients [25]. The present data only indicate that, within the observed range and after adjustment for other factors, image noise did not add significant independent information.

The results may help readers recognize segments at greater risk of incomplete interpretation on non-ECG-gated CCTA. Heavily calcified, motion-prone, or small-caliber segments may require further evaluation when clinically important disease cannot be excluded. However, the study does not show that non-ECG-gated CCTA can replace conventional ECG-gated CCTA or define a clinical use boundary without an independent accuracy reference standard. Several limitations require consideration. This was a retrospective single-center study using one vendor-specific scanner platform, phase-selection workflow, motion-correction algorithm, and reconstruction approach; the findings therefore should not be assumed to generalize directly to other CT platforms or motion-correction implementations. No eligible ICA or ECG-gated CCTA reference-standard subgroup was available for the prespecified diagnostic-accuracy analysis, precluding assessment of diagnostic accuracy or direct comparison with conventional ECG-gated CCTA. The study-specific visual calcification scale limits comparison with established quantitative calcium categories. Calcification severity and evaluability were assessed by the same readers on the same contrast-enhanced CCTA datasets. Although separate criteria were used, calcium blooming could influence both perceived calcification extent and lumen visibility, potentially introducing correlated measurement error or differential misclassification. Stratified severe-calcification subgroups were small and descriptive. In addition, 16 globally non-diagnostic examinations were excluded from the primary segment-level analysis, which may have inflated the primary evaluability estimate; however, sensitivity analysis incorporating their 182 observed eligible segments as non-evaluable reduced overall evaluability to 86.2%, and an 18-segment worst-case boundary yielded 83.9%. Prospective multicenter validation with an independent reference standard is therefore needed.

## 5. Conclusions

In conclusion, most included coronary segments were evaluable on dedicated non-ECG-gated CCTA, but evaluability varied by calcification severity, heart rate, coronary territory, and vessel diameter. These findings describe segment-level feasibility in this retrospective single-center cohort. Diagnostic accuracy, equivalence to ECG-gated CCTA, and clinically actionable use thresholds require prospective validation against an independent reference standard.

## Figures and Tables

**Table 1:** Demographic, clinical, and imaging characteristics.

Characteristic	Value
Patients, *n*	320
Age, years	60.7 ± 10.1
Male sex, *n* (%)	183 (57.2)
BMI, kg/m^2^	25.1 ± 3.2
Heart rate, beats/min	72.9 ± 11.3
Rhythm, *n* (%)	
Sinus rhythm	293 (91.6)
Atrial fibrillation	15 (4.7)
Frequent ectopy	12 (3.8)
Hypertension, *n* (%)	140 (43.8)
Diabetes mellitus, *n* (%)	57 (17.8)
Current/former smoking, *n* (%)	90 (28.1)
Height, cm	167.1 ± 7.5
Body weight, kg	70.1 ± 10.8
Contrast volume, mL	42.1 ± 6.4
Injection rate, mL/s	4.21 ± 0.64
Tube voltage, *n* (%)	
80 kV	52 (16.3)
100 kV	221 (69.1)
120 kV	47 (14.7)
CTDIvol, mGy	4.4 ± 0.9
Aortic root attenuation, HU	411 ± 47
Image noise, HU	18.5 ± 2.5

Values are mean ± standard deviation or *n* (%). BMI, body mass index; CTDIvol, volume CT dose index; HU, Hounsfield unit.

**Table 2:** Segment evaluability by calcification severity.

Calcification	Total, *n*	Evaluable, *n* (%)	Non-Evaluable, *n* (%)
None	2146	2004 (93.4)	142 (6.6)
Mild	1058	940 (88.8)	118 (11.2)
Moderate	364	307 (84.3)	57 (15.7)
Severe	171	127 (74.3)	44 (25.7)
Overall	3739	3378 (90.3)	361 (9.7)

**Table 3:** Segment evaluability by coronary territory and anatomical location.

Category	Total, *n*	Evaluable, *n* (%)	Non-Evaluable, *n* (%)
LM	320	301 (94.1)	19 (5.9)
LAD	1183	1085 (91.7)	98 (8.3)
LCX	851	774 (91.0)	77 (9.0)
RCA	1385	1218 (87.9)	167 (12.1)
Proximal/mid	1920	1775 (92.4)	145 (7.6)
Distal/branch	1819	1603 (88.1)	216 (11.9)

LM, left main; LAD, left anterior descending; LCX, left circumflex; RCA, right coronary artery.

## Data Availability

The de-identified data that support the findings of this study and the analysis code are available from the corresponding author upon reasonable request, subject to institutional and ethical requirements.

## Supplementary Materials

The following supporting information can be downloaded at https://www.mdpi.com/article/10.3390/diagnostics16193234/s1, Table S1. Patient- and vessel-level evaluability. Table S2. Segment evaluability according to pre-acquisition heart-rate category. Table S3. Sensitivity analyses. Table S4. Motion artifact, subjective image quality, failure reasons, and reader-specific evaluability. Table S5. Rhythm-stratified segment evaluability and characteristics of globally non-diagnostic examinations. Figure S1. Segment evaluability according to pre-acquisition heart-rate category. Error bars represent patient-level cluster-bootstrap 95% confidence intervals. Figure S2. Sensitivity GEE model excluding reference vessel diameter. Points are adjusted odds ratios and horizontal bars are 95% confidence intervals. The vertical line indicates an odds ratio of 1.0. Figure S3. Overall distribution of coronary motion-artifact grades across 3739 included segments. Figure S4. Overall distribution of subjective image-quality scores across 3739 included segments.
