# Supplementary material for: Segment-Level Evaluability of Dedicated Non-ECG-Gated Coronary CT Angiography: Associations with Calcification, Heart Rate, Vessel Territory, and Caliber
Source: Diagnostics (Basel). 2026 Oct 7;16(19):3234. doi: 10.3390/diagnostics16193234 (PMC13664505; doi:10.3390/diagnostics16193234)
Supplement: Supplementary file 1 [file diagnostics-16-03234-s001.zip › diagnostics-4591803-supplementary.pdf]

Supplementary Material

Segment-Level Evaluability of Dedicated Non-ECG-Gated Coronary CT Angiography: Associations with Calcification, Heart Rate, Vessel Territory, and Caliber

Table S1. Patient- and vessel-level evaluability.

Endpoint	n/N	%	95% CI, if applicable
All included coronary segments evaluable	118/320	36.9	31.6–42.2
≥1 non-evaluable coronary segment	202/320	63.1	57.8–68.4
≥1 non-evaluable proximal/mid segment	111/320	34.7	29.4–40.0
All LM segments evaluable	301/320	94.1	—
All LAD segments evaluable	243/320	75.9	—
All LCX segments evaluable	254/320	79.4	—
All RCA segments evaluable	192/320	60.0	—
Sensitivity analysis including globally non-diagnostic examinations			
All included coronary segments evaluable	118/336	35.1	—
≥1 non-evaluable coronary segment	218/336	64.9	—
≥1 non-evaluable proximal/mid segment	127/336	37.8	—

Patient-level confidence intervals were obtained by patient-level bootstrap resampling. LM, left main; LAD, left anterior descending; LCX, left circumflex; RCA, right coronary artery.

Table S2. Segment evaluability according to pre-acquisition heart-rate category.

Heart-rate category	Patients, n	Segments, n	Evaluable, n/N (%)	Cluster-bootstrap 95% CI
<65 beats/min	84	987	922/987 (93.4)	91.9–94.8
65–80 beats/min	162	1889	1726/1889 (91.4)	90.0–92.8
>80 beats/min	74	863	730/863 (84.6)	81.6–87.3

Confidence intervals account for within-patient clustering by patient-level bootstrap resampling.

Table S3. Sensitivity analyses.

Analysis	Estimate	95% CI / P value
Examination success with 16 globally non-diagnostic examinations counted as failures	320/336 (95.2%)	92.4–97.0%
Observed-failure segment sensitivity: 182 eligible segments from 16 globally non-diagnostic examinations included as non-evaluable	3378/3921 (86.2%)	85.0–87.2%
Conservative segment-level boundary: 18 non-evaluable SCCT segments assigned to each of 16 failed examinations	3378/4027 (83.9%)	82.7–85.0%
Quadratic heart-rate term added to clustered GEE	No evidence of departure from linearity	P=0.177
GEE without reference diameter: mild vs none	OR 1.96 (1.47–2.61)	P<0.001
GEE without reference diameter: moderate vs none	OR 3.14 (2.19–4.50)	P<0.001
GEE without reference diameter: severe vs	OR 6.64 (4.46–9.87)	P<0.001

Analysis	Estimate	95% CI / P value
none		
GEE without reference diameter: HR per 10 beats/min	OR 1.52 (1.36–1.69)	P<0.001
GEE without reference diameter: BMI per 5 kg/m²	OR 0.84 (0.69–1.01)	P=0.069
GEE without reference diameter: distal/branch vs proximal-mid	OR 1.85 (1.46–2.35)	P<0.001
GEE without reference diameter: LCX vs LAD/LM	OR 1.06 (0.76–1.46)	P=0.747
GEE without reference diameter: RCA vs LAD/LM	OR 1.56 (1.19–2.02)	P=0.001
GEE without reference diameter: image noise per HU	OR 1.01 (0.97–1.06)	P=0.518

The 86.2% estimate incorporates the 182 observed eligible coronary segments from the 16 globally non-diagnostic examinations as non-evaluable. The 83.9% estimate is an additional deliberately conservative boundary assigning all 18 SCCT segments as non-evaluable to each failed examination.

Figure S1. Segment evaluability according to pre-acquisition heart-rate category. Error bars represent patient-level cluster-bootstrap 95% confidence intervals.

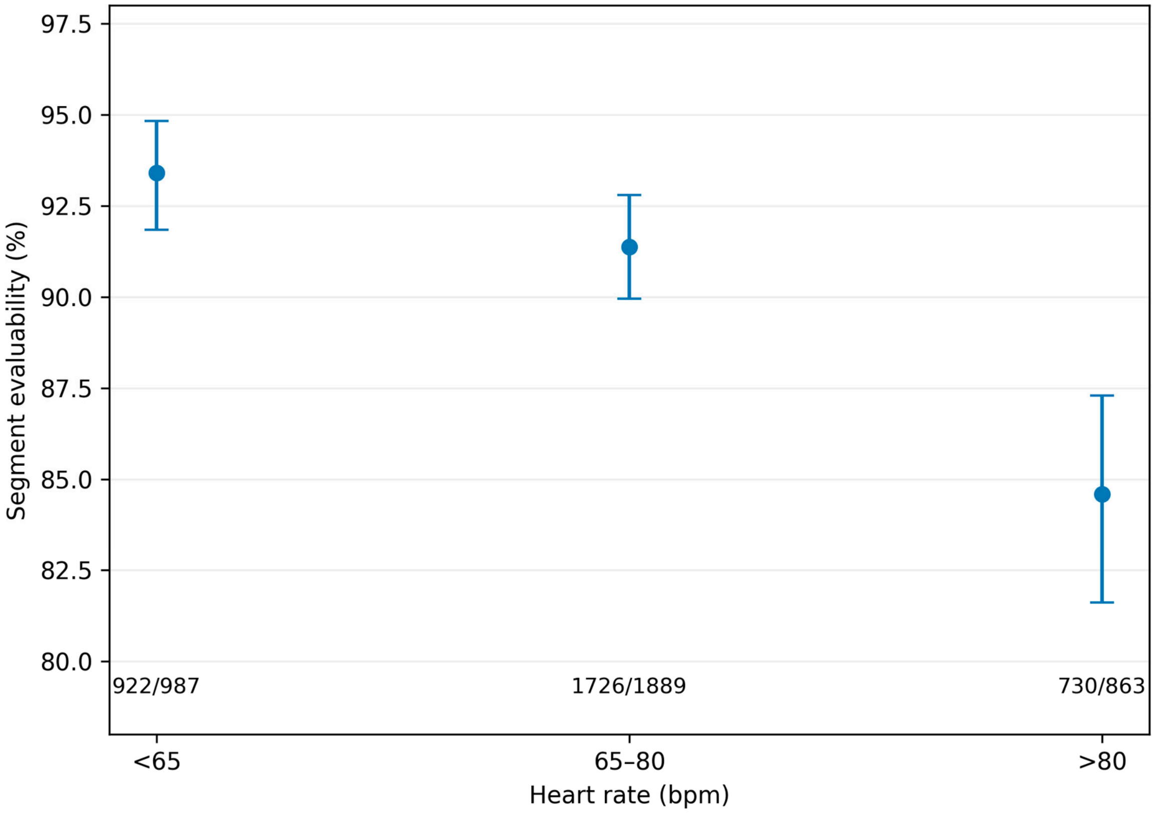

Figure S2. Sensitivity GEE model excluding reference vessel diameter. Points are adjusted odds ratios and horizontal bars are 95% confidence intervals. The vertical line indicates an odds ratio of 1.0.

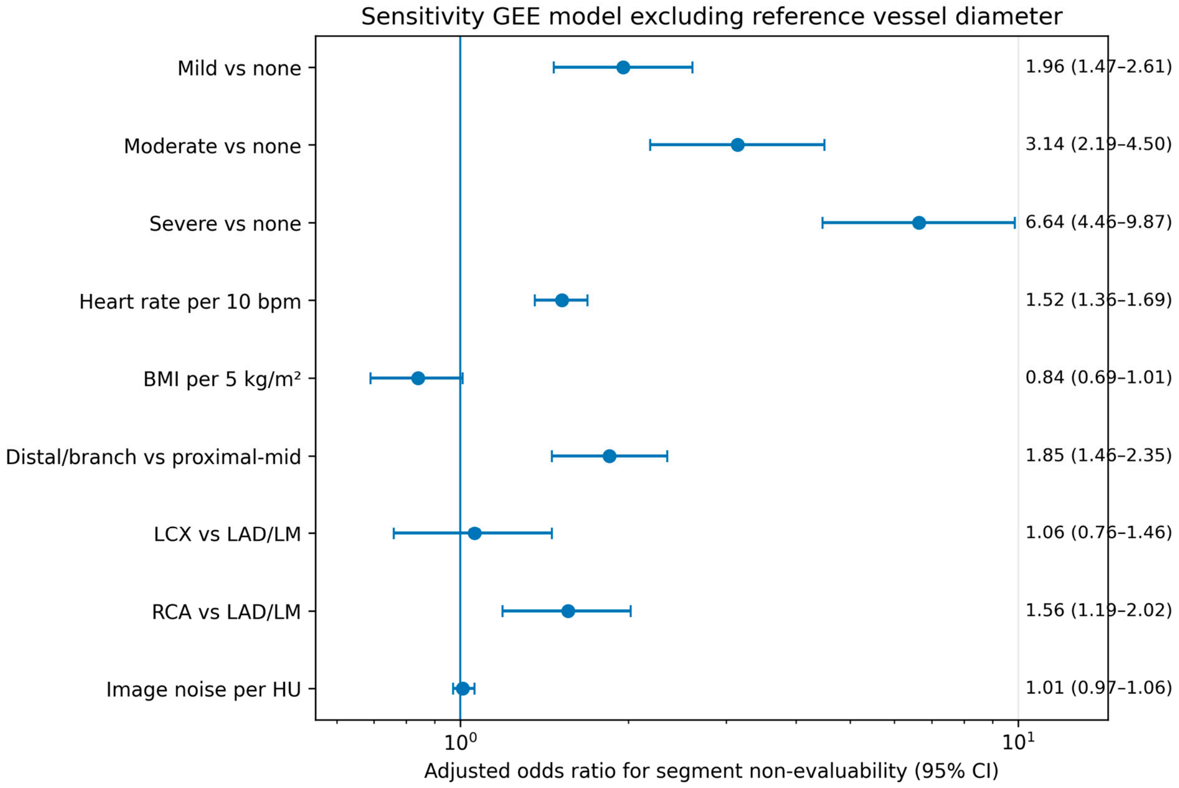

Table S4. Motion artifact, subjective image quality, failure reasons, and reader-specific evaluability.

A. Overall motion-artifact and subjective image-quality distributions.

Measure	Category	n	%
Motion grade	0	1952	52.2
Motion grade	1	1139	30.5
Motion grade	2	450	12.0
Motion grade	3	198	5.3
Subjective image quality	4	1530	40.9
Subjective image quality	3	1588	42.5
Subjective image quality	2	443	11.8
Subjective image quality	1	178	4.8

B. Primary reason for non-evaluability among 361 non-evaluable segments.

Primary reason	n	%
Motion artifact	202	56.0
Calcification/blooming	107	29.6
Insufficient enhancement	22	6.1
Excessive image noise	18	5.0
Other artifact	12	3.3

C. Reader-specific binary evaluability results.

Metric	Result
Reader 1 non-evaluable	379/3739 (10.1%)
Reader 2 non-evaluable	386/3739 (10.3%)
Simple agreement	98.4%
Consensus adjudication	59/3739 (1.6%)
Cohen's κ	0.914 (patient-cluster bootstrap 95% CI, 0.890–0.935)

D. Motion-grade and subjective image-quality distributions by coronary territory, n (%).

Territory	Segments	Motion 0	Motion 1	Motion 2	Motion 3	IQ 4	IQ 3	IQ 2	IQ 1
LM	320	175 (54.7)	99 (30.9)	33 (10.3)	13 (4.1)	146 (45.6)	125 (39.1)	36 (11.2)	13 (4.1)
LAD	1183	637 (53.8)	353 (29.8)	140 (11.8)	53 (4.5)	472 (39.9)	506 (42.8)	149 (12.6)	56 (4.7)
LCX	851	437 (51.4)	269 (31.6)	100 (11.8)	45 (5.3)	361 (42.4)	365 (42.9)	85 (10.0)	40 (4.7)
RCA	1385	703 (50.8)	418 (30.2)	177 (12.8)	87 (6.3)	551 (39.8)	592 (42.7)	173 (12.5)	69 (5.0)

E. Motion-grade and subjective image-quality distributions by pre-acquisition heart-rate category, n (%).

Heart-rate category	Segments	Motion 0	Motion 1	Motion 2	Motion 3	IQ 4	IQ 3	IQ 2	IQ 1
<65 beats/min	987	521 (52.8)	322 (32.6)	113 (11.4)	31 (3.1)	429 (43.5)	413 (41.8)	114 (11.6)	31 (3.1)
65–80 beats/min	1889	993 (52.6)	588 (31.1)	211 (11.2)	97 (5.1)	789 (41.8)	813 (43.0)	195 (10.3)	92 (4.9)
>80 beats/min	863	438 (50.8)	229 (26.5)	126 (14.6)	70 (8.1)	312 (36.2)	362 (41.9)	134 (15.5)	55 (6.4)

Motion grade: 0, none; 1, mild; 2, moderate; 3, marked. Subjective image quality: 4, excellent; 3, good; 2, fair; 1, poor. Percentages are calculated within the corresponding row denominator.

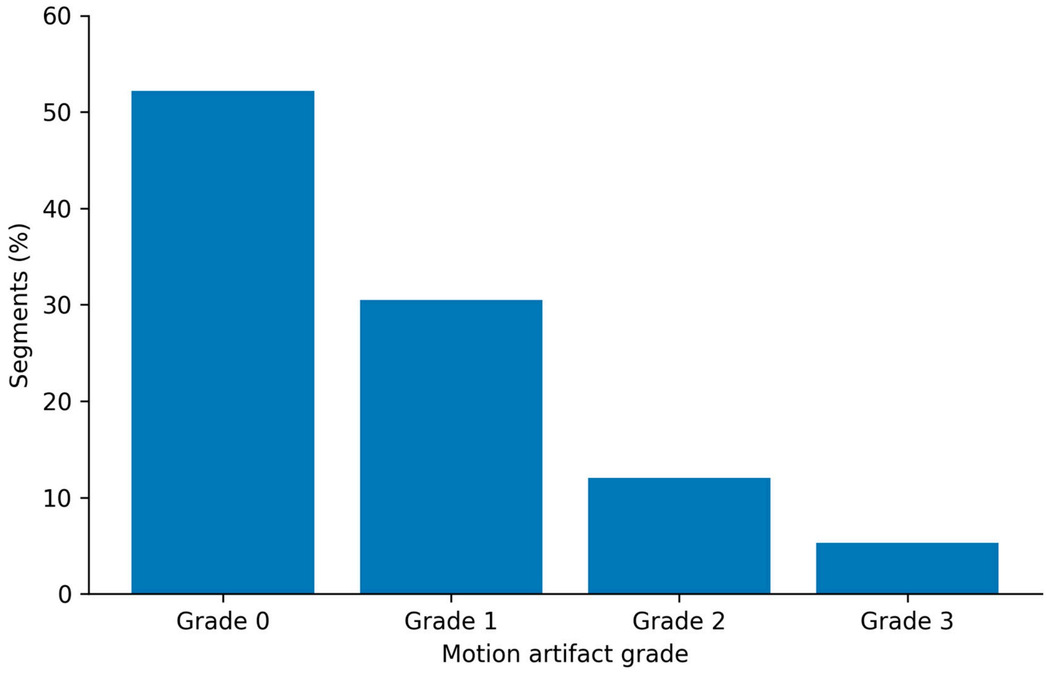

Figure S3. Overall distribution of coronary motion-artifact grades across 3739 included segments.

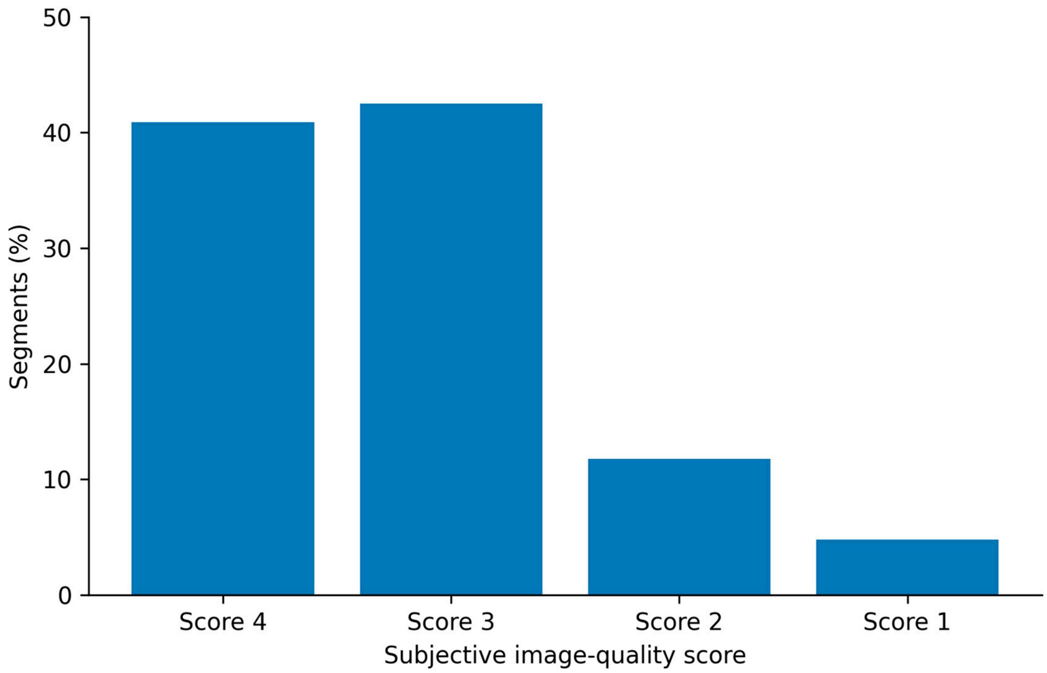

Figure S4. Overall distribution of subjective image-quality scores across 3739 included segments.

Table S5. Rhythm-stratified segment evaluability and characteristics of globally non-diagnostic examinations.

Rhythm in primary cohort	Patients, n	%
Sinus rhythm	293	91.6
Atrial fibrillation	15	4.7
Frequent ectopy	12	3.8
Characteristic of globally non-diagnostic examinations	Patients, n	%
Sinus rhythm	12	75.0
Atrial fibrillation	3	18.8
Frequent ectopy	1	6.3
Severe respiratory/bulk motion	9	56.3
Inadequate contrast enhancement	5	31.3
Major reconstruction failure	2	12.5
Primary-cohort segment evaluability: sinus rhythm	3085/3421	90.2
Primary-cohort segment evaluability: atrial fibrillation	159/175	90.9
Primary-cohort segment evaluability: frequent ectopy	134/143	93.7
Global examination failure: sinus rhythm	12/305	3.9
Global examination failure: atrial fibrillation	3/18	16.7
Global examination failure: frequent ectopy	1/13	7.7

The 16 globally non-diagnostic examinations contributed 182 observed eligible coronary segments: 95 were from examinations failing because of severe respiratory/bulk motion, 66 because of inadequate contrast enhancement, and 21 because of major reconstruction failure. All 182 segments were classified as non-evaluable in the observed-failure sensitivity analysis.

Among the three globally non-diagnostic examinations in patients with atrial fibrillation, two failed because of severe respiratory/bulk motion and one because of inadequate contrast enhancement.
